# Wheat seed weight and quality differ temporally in sensitivity to warm or cool conditions during seed development and maturation

**DOI:** 10.1093/aob/mcx074

**Published:** 2017-06-15

**Authors:** M Nasehzadeh, R H Ellis

**Affiliations:** 1 School of Agriculture, Policy and Development, University of Reading, Earley Gate, PO Box 237, Reading RG6 6AR, UK

**Keywords:** Bread-making quality, climate change, seed desiccation, seed development, seed filling, seed weight, seed germination, seed longevity, temperature, wheat, *Triticum aestivum* L

## Abstract

**Background and aims:**

Short periods of extreme temperature may affect wheat (*Triticum aestivum*) seed weight, but also quality. Temporal sensitivity to extreme temperature during seed development and maturation was investigated.

**Methods:**

Plants of ‘Tybalt’ grown at ambient temperature were moved to growth cabinets at 29/20°C or 34/20°C (2010), or 15/10°C or 34/20°C (2011), for successive 7-d periods from 7 DAA (days after anthesis) onwards, and also 7–65 DAA in 2011. Seed samples were harvested serially and moisture content, weight, ability to germinate, subsequent longevity in air-dry storage and bread-making quality were determined.

**Key Results:**

High temperature (34/20°C) reduced final seed weight, with greatest temporal sensitivity at 7–14 or 14–21 DAA. Several aspects of bread-making quality were also most sensitive to high temperature then, but whereas protein quality decreased protein and sulphur concentrations improved. Early exposure to high temperature provided earlier development of ability to germinate and tolerate desiccation, but had little effect on maximum germination capacity. All treatments at 15/10°C resulted in ability to germinate declining between 58 and 65 DAA. Early exposure to high temperature hastened improvement in seed storage longevity, but the subsequent decline in late maturation preceded that in the control. Long (7–65 DAA) exposure to 15/10°C disrupted the development of seed longevity, with no improvement after seed filling ended. Longevity improved during maturation drying in other treatments. Early (7–14 DAA) exposure to high temperature reduced and low temperature increased subsequent longevity at harvest maturity, whereas late (35 or 42–49 DAA) exposure to high temperature increased and low temperature reduced it.

**Conclusions:**

Temporal sensitivity to extreme temperature was detected. It varied considerably amongst the contrasting seed variables investigated. Subsequent seed longevity at harvest maturity responded negatively to temperature early in development, but positively later in development and throughout maturation.

## INTRODUCTION

Wheat (*Triticum aestivum*) is a common crop of temperate regions but is also grown in the tropics and subtropics (Klatt, 1988) with grain production across wide ranges of altitude (sea level to 3500 m) and latitude (60°N to 60°S) (Curtis, 2015). Bread from wheat is a major human food (Bordes *et al.*, 2008). The 2002–2011 decadal average European temperature was 1·3°C greater than 1850–1899 (IPCC, 2014), whilst various climate change scenarios suggest a 2·0–4·0°C warming towards the end of this century (IPCC, 2013). For many annual crops, yield is affected not only by mean change in temperature but also by brief periods of high temperature at vulnerable stages of development (Wheeler *et al.*, 2000; Rezaei *et al.*, 2015; Prasad *et al.*, 2017). Future climates will provide more frequent high temperature extremes of longer duration, but occasional cold winter extremes will continue (IPCC, 2013). The impacts of short periods of extreme temperature on wheat seed development and quality are therefore relevant to the global adaptation of the crop today – and even more to adaptation to future climates. Whilst the effect of temperature variability at early stages of reproductive development on yield is becoming better understood, quality might also show temporal sensitivity to extreme temperature. In the case of wheat, poor-quality crops may be rejected as being unsuitable for making bread or to sow to establish subsequent crops.

Seed set, and hence yield, in wheat is greatly reduced by temperatures above 30 or 31°C around anthesis even with only brief exposure (Wheeler *et al.*, 1996; Ferris *et al.*, 1998) and at both meiosis and anthesis after only 1 d at 35/30°C (Barber *et al.*, 2017). Higher temperatures post-anthesis in wheat increase the rate but reduce the duration of seed filling (Sofield *et al.*, 1977; Shpiler and Blum, 1990; Motzo *et al.*, 2010; Cossani and Reynolds, 2012; Kobata *et al.*, 2012). Final seed dry weight is the product of rate and duration of filling (Gallagher *et al.*, 1976). The latter has the greater effect on seed weight. A day temperature of 25°C or less from anthesis onwards has been reported to be optimal for seed filling in wheat (Feyerherm and Paulsen, 1981; Porter and Gawith, 1999), but seed dry weight is reduced if average temperature exceeds 15°C (Sofield *et al.*, 1977; Al-Khatib and Paulsen, 1984; Slafer and Rawson, 1994). Some wheat-producing areas, however, have ambient temperatures of 37–40°C during grain-filling (Marcellos and Single, 1972). Even in temperate regions maximum temperatures may approach such values briefly and the timing of high temperature events during seed filling is important for both grain yield and quality (Stone and Nicolas, 1996).

Development of the ability to germinate in wheat improves with higher temperature during seed filling and maturation (Nicholls, 1980; Sanhewe *et al.*, 1996), although not always consistently so (Grass and Burris, 1995). The more rapid development of seeds at higher temperatures results in more rapid seed desiccation which may increase subsequent rate of germination (Wellington, 1956). Seed dormancy may also be affected; seeds which develop in cooler conditions tend to show greater dormancy (Black *et al.*, 1987; Walker-Simmons, 1987). Seed quality in wheat, as assessed by subsequent seed longevity (period for the viability of the population to decline to a given value) in air-dry storage not only increases during seed filling until the end of that phase, termed mass maturity (Ellis and Pieta Filho, 1992), but also during subsequent maturation drying until close to harvest maturity (Ellis and Pieta Filho, 1992; Sanhewe *et al.*, 1996; Ellis and Yadav, 2016; Yadav and Ellis, 2016). (Harvest maturity in a cereal crop is the end of natural seed desiccation when seed moisture content is in dynamic equilibrium with ambient relative humidity.) The rate of improvement in subsequent seed longevity was positively associated with post-anthesis temperature (Sanhewe *et al.*, 1996). 

Variation in the properties of wheat flour is a major problem in making bread and so affects the crop’s value (Kennett *et al.*, 1998). To make bread, wheat should have high protein concentration and quality (indicated by high sulphur concentration), high sodium dodecyl sulphate (SDS) sedimentation volume and high Hagberg Falling number (HFN) (Gooding and Davies, 1997). Bread-making quality depends upon genotype, but is also affected greatly by environment (Graybosch *et al.*, 1996; Huebner *et al.*, 1997; Zhu and Khan, 2001). Temperature affects protein concentration, starch properties and dough strength (Evans *et al.*, 1975; Randall and Moss, 1990; Tester *et al.*, 1995; Daniel and Triboi, 2002). Wheat grain protein concentration, for example, can range from 8 to 20 % depending upon genotype and environment (Wieser and Kieffer, 2001).

Here, we investigate the effect of short periods of extreme temperature post-anthesis on wheat seed filling, maturation, development of ability to germinate, subsequent seed storage longevity and bread-making quality in order to identify the most sensitive period to extreme temperature. The null hypotheses were that short periods of extreme temperature at different stages during seed development and maturation had no effect on seed filling, ability to germinate, subsequent seed storage longevity or bread-making quality. The objective was to determine whether a short period of extreme temperature affected each of these parameters and, if so, similarly or not, and to identify potential temporal variation in sensitivity during seed and seed quality development.

## MATERIAL AND METHODS

Two investigations were carried out at the Plant Environment Laboratory (51°27′N, 00°94′W) in modified and controlled environments in successive years. Seven seeds of wheat (*Triticum aestivum* L.) ‘Tybalt’ were sown 2cm deep into each pot (180mm diameter, 3-litre capacity, 700 pots in total in 2010 and 934 in 2011) and thinned 19 d (2010) to 23 d (2011) later to provide four strong seedlings per pot (a plant population density of approx. 123 plants m^−2^). The previously steam-sterilized growing medium comprised vermiculite, sand, gravel and loam-less peat compost (Sinclair Potting and Bedding Compost, JFC Monro, Hayle, UK), 4 : 2 : 4 : 1, mixed with Osmocote (Osmocote Exact-Scotts, Everris International B.V., Geldermalsen, The Netherlands) slow-release granules (2kg m^−3^) containing N/P_2_O_5_/K_2_O/MgO (15 : 11 : 13 : 2). Pots were arranged randomly, in terms of later treatments identified at the outset, in the central area of a polythene tunnel house (31 ×8 m) in which fan-assisted ventilation was provided to reduce solar gain in order to provide temperatures close to ambient. Guard rows surrounded the pots destined for experimental treatment. An automated drip-feed system provided irrigation from seedling emergence onwards once a day initially, and then twice a day. Plants were staked at anthesis to avoid damage when pots were subsequently moved.

A control (‘ambient’) treatment was maintained in this tunnel house environment throughout. The remaining pots were moved at different dates from 7 DAA (days after 50 % anthesis) onwards for successive 7-d periods at different temperatures in modified Saxcil plant growth cabinets (internal dimensions 1·4 × 1·4 × 1·5 m) (48 pots per cabinet). Carbon dioxide in the cabinets was maintained at 385μmol mol^−1^ of air, relative humidity at 55±10 % (day) and 85±10 % (night), and a photosynthetic photon flux density of 650μmol m^−2^ s^−1^ (from cool white fluorescent tubes and incandescent lamps). After 7 d, the pots were returned to the tunnel house.

### Treatments, Experiment 1, 2010

Seeds were sown on 14 April 2010. The six treatments comprised warmer regimes of 29/20°C or 34/20°C (16/8h d^−1^) for 7 d at 7–14 (T_1_), 14–21 (T_2_), 21–28 (T_3_), 28–35 (T_4_), 35–42 (T_5_) or 42–49 DAA (T_6_), before pots were returned to ambient. Each treatment combination comprised up to 48 pots (fewer pots were required for later treatments). Samples from each treatment combination, once treatment had begun, were harvested destructively up to seven times at 7-d intervals at 19, 26, 33, 40, 47, 54 or 61 DAA (5 July to 17 August). Harvests did not coincide precisely with treatment end dates and extended beyond the last of these (in both years) because the effect of treatments on seeds was studied throughout their subsequent development and maturation.

### Treatments, Experiment 2, 2011

Seeds were sown on 14 April 2011. Six 7-d treatments at conditions cooler or warmer than ambient [15/10 or 34/20°C (16/8h d^−1^), respectively] were provided from 7 DAA, as Experiment 1. In addition, two longer-duration treatments were provided in additional cabinets: 7–65 DAA at 15/10 or 34/20°C. Samples from each treatment combination, once treatment had begun, were harvested destructively up to eight times at 7-d intervals at 16, 23, 30, 37, 44, 51, 58 or 65 DAA (30 June to 19 August).

### Seed harvest, weight and moisture content

Harvests were taken at 0800–1000 h, seeds detached from ears carefully by hand, and fresh seed samples drawn to determine weight, moisture content and ability to germinate. Additional samples were harvested 12 (2010) or 9 (2011) DAA to monitor early seed development. Since seed number is determined at anthesis, the effects of treatments thereafter on seed weight are comparable to those on yield. The remaining seeds were dried to 10–14 % moisture content (fresh weight basis) in a drying cabinet (15–17°C with 12–15 % relative humidity), a further sample was then drawn to determine ability to germinate, and the remainder were stored hermetically at−20°C until the determination of longevity.

Two additional pots were selected randomly from each treatment and harvested at 61 DAA in 2010 to estimate harvest index (proportion of seed dry weight to total above-ground dry weight). The plants were cut at the soil surface, separated into ears and straw, and seeds dissected out by hand. The fresh weight of chaff, straw and seed was determined immediately and dry weight of each component determined after drying in a forced-air oven at 80°C for 48h. Additional samples were also drawn from each treatment in the final harvest each year (61 DAA, 2010; 65 DAA, 2011) to analyse bread-making quality.

Moisture content was determined by the high-constant temperature oven method at 130°C and calculated as a percentage of fresh seed weight (ISTA, 2013). The two-stage method was used for seed moisture contents >17 % (ISTA, 2013). Two replicates of 500 seeds were sampled, counted and weighed to determine mean seed fresh weight. Mean seed dry weight was calculated from these estimates of fresh weight and moisture content.

### Ability to germinate

Freshly harvested or dried (<15 % moisture content) seeds were tested for ability to germinate between rolled paper towels (Kimberley Clark Professional 6803 Hostess, Natural, 24×35cm, Greenham Sales, UK), moistened with deionized water, in an incubator at 10°C for 28 d, initially, with samples of 100 seeds divided between two replicates. Seeds remaining un-germinated and fresh after 28 d were then pricked and returned to the test until all seeds had either germinated or rotted. Seedlings were evaluated for normal development (ISTA, 2013).

### Air-dry seed storage longevity

Seed samples withdrawn from temporary storage at−20°C were kept sealed overnight at 20°C to warm before opening the packets. The moisture content of each sample was adjusted to 15±0·2 % by humidification above water at 20°C (for 2–24h, depending on initial moisture content), and samples were then stored hermetically at 3–5°C for 5 d to allow moisture to equilibrate. For each seed lot, 10–11 samples of 110 seeds were sealed in separate laminated-aluminium-foil bags (Retort laminate, Moore and Buckle Ltd, St Helens, UK). These bags were then stored in an incubator maintained at 40°C (±0·5°C). Samples were removed from experimental storage to test ability to germinate (as above) at regular intervals for up to 46 d. The criterion of seed survival was ability to produce a normal seedling (ISTA, 2013).

### Bread-making quality

Dried samples from final harvests (61 DAA, 2010; 65 DAA, 2011) were stored hermetically (2–5°C with approx. 14% moisture content) and then milled with a Laboratory Mill 3100 (Perten Instruments AB, Huddinge, Sweden). Seed nitrogen and sulphur (S) concentrations were determined for two replicates of a 0·2-g sample of dried wholemeal flour by the oxidative combustion method (AOACI, 2005) using automated Dumas type combustion analysers [LECO FP-528 and LECO SC-144DR, LECO Instruments (UK) Ltd, Stockport, UK]. Crude protein concentration (% DM) was calculated by multiplying nitrogen (%) by 5·7 (Gooding and Davies, 1997). SDS sedimentation volume was estimated (Axford *et al.*, 1979): 6g of wholemeal wheat flour samples (two per treatment combination) at 15 % moisture content were suspended in 50mL distilled water in a 100-mL cylinder, 50mL SDS reagent (stock solution: 20g SDS with 20mL diluted lactic acid in 1litre of distilled water) added and shaken well to form a suspension, allowed to settle for 20min, and sediment volume recorded. HFN was estimated with equipment incorporating automatic agitation (Falling Number 1500; ISO 3093: BSI, 1982; Perten Instruments, Sweden). Each sample (two per treatment combination) of 7g flour at 15 % moisture content was first suspended in 25mL distilled water.

### Data analysis

Genstat 14 (VSN International Ltd, Hemel Hempstead, UK) was used for all analyses. Preliminary analysis of variance (ANOVA) was applied to each data set to assess treatment effects, with results for percentage germination first arcsine transformed. The progress of seed filling [DW = mean seed dry weight (mg) versus DAA] was curvilinear and so the ordinary logistic regression model

DW=A+C/[1+ exp  (−B* (DAA−M))] (1)

was applied to quantify this where *C* is final dry weight (mg), *B* rate of growth (slope), *M* is an inflection point for time (DAA) and *A* is the intercept at zero time (set to zero because seed filling had not begun at anthesis) (Zahedi and Jenner, 2003). aMaximum seed filling rate (*R*_max_) and duration from 50 % anthesis to 95 % final seed dry weight (T_95_) were calculated as

$$R_{\text{max}}=\left(B*C\right)/4$$ (2)

$$T_{95}=\left(B*M+2.944\right))/B$$ (3)

(Zahedi and Jenner, 2003). Mass maturity, defined as the end of the seed-filling phase (Ellis and Pieta Filho, 1992), is quantified by T_95_ here therefore.

Seed survival curves were fitted to observations of ability of seeds to germinate normally stored for different periods by probit analysis in accordance with the seed viability equation

$$v=K_{i}–p/\sigma$$ (4)

where *v* is probit percentage viability after *p* days in storage, *K_i_* is a constant (equivalent to probit percentage viability at start of experimental storage) and *σ* is the standard deviation of the frequency distribution of seed deaths in time (d) (Ellis and Roberts, 1980). The product of *K*_i_ and *σ* is the period (d) for viability to decline to 50 % (*p*_50_). 

## RESULTS

Post-anthesis mean temperature in the polythene-tunnel house was 1·1°C higher in 2010 (19·7°C) than 2011 (18·6°C). The latter provided a greater mean diurnal range. Temperature ranged between extremes of 7·7 and 33·7°C, with mean daily minima and maxima of 13·4 and 28·7°C in 2010. Hence the 2010 ambient control regime had a maximum (day) temperature close to that of the cooler cabinet environment, but a minimum (night) temperature some 6·6°C lower. In 2011, temperatures in the ambient control ranged from 7·3 to 37·1°C, with mean daily minima and maxima of 11·1 and 29·3°C. Nominal temperatures in the cabinets were maintained to within ±1°C. 

### Seed desiccation

**Fig. 1.**
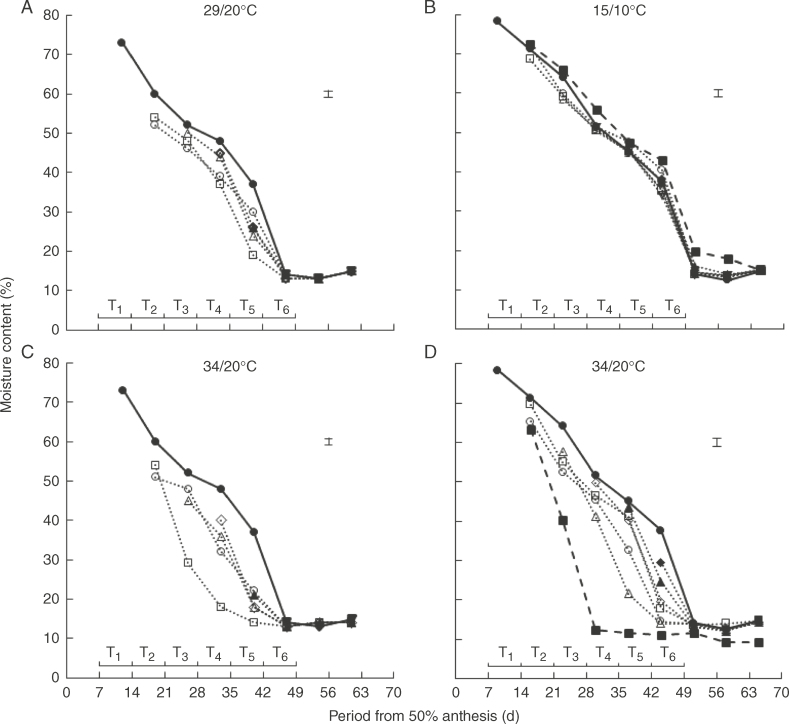
The relationship between seed moisture content (%, fresh weight basis) and period from anthesis (d) in spring wheat ‘Tybalt’ control plants maintained at close to ambient temperature throughout (**•**) or exposed for different, successive 7-d periods from 7 DAA onwards to day/night temperatures of (A) 29/20°C and (C) 34/20°C (2010) or (B) 15/10°C and (D) 34/20°C (2011); T_1_ (○), T_2_ (□), T_3_ (△), T_4_ (◊), T_5_ (▲), T_6_ (◆). In 2011, treatments were also provided at 15/10°C or 34/20°C from 7–65 DAA (◼). The horizontal bars represent the different 7-d periods (T_1_–T_6_) when temperatures higher or lower than ambient were applied; vertical bars are least significant difference (*P* = 0·05).

The six temperature treatment periods T_1_ to T_6_ spanned the period of seed desiccation well: final (dynamic equilibrium) moisture content (harvest maturity) in the control reached 2 d before or 2 d after T_6_ ended in 2010 and 2011, respectively (Fig. 1). Temperature, time of treatment, time of harvest, and all first- and second-order interactions amongst them affected seed moisture content (*P*<0·001) in each year. The progress of desiccation was advanced at 29/20°C and yet more so at 34/20°C, with T_2_ providing the most rapid and the ambient control the slowest desiccation in 2010 (Fig. 1A, C). In 2011, 7-d treatments at 15/10°C had little effect whilst the 7–65 DAA treatment provided only a slight delay to desiccation generally, except for a pronounced delay to final drying from 51 to 65 DAA (Fig. 1B); at 34/20°C all seven treatments resulted in more rapid desiccation than the control, with the 7–65 DAA treatment drying to harvest maturity well before the 7-d treatments (Fig. 1D) and desiccation period ranked T_3_ < T_1_ < T_2_ = T_4_ < T_5_ < T_6_ amongst the latter.

### Seed filling

**Fig. 2.**
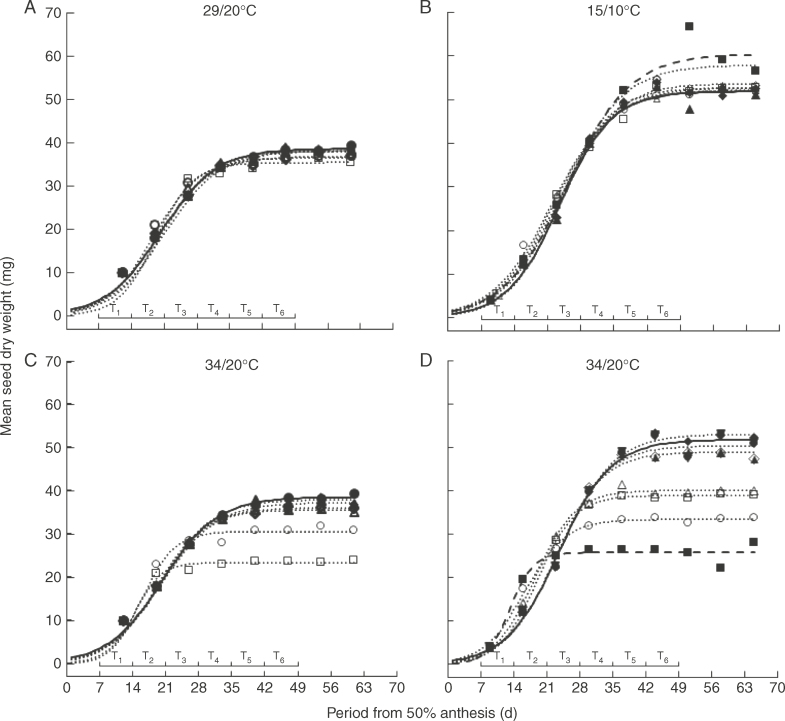
Changes in mean seed dry weight (mg) during seed development in spring wheat ‘Tybalt’ control plants maintained at close to ambient temperature throughout (**•**) or exposed for different, successive 7-d periods from 7 DAA onwards to day/night temperatures of (A) 29/20°C and (C) 34/20°C (2010) or (B) 15/10°C and (D) 34/20°C (2011); T_1_ (○), T_2_ (□), T_3_ (△), T_4_ (◇), T_5_ (▲), T_6_ (◆). In 2011, treatments were also provided at 15/10°C or 34/20°C from 7 to 65 DAA (◼). The horizontal bars represent the different 7-d periods (T_1_–T_6_) when temperatures higher or lower than ambient were applied. The fitted curves are quantified in Table 1.

Temperature, time of treatment, time of harvest, and all first- and second-order interactions amongst them affected seed dry weight (*P*<0·001) in each year. Seed filling was described well by logistic regressions (Fig. 2, Table 1), which differed amongst treatments (*P* < 0·05). Final seed weight in ambient controls was greater in the cooler 2011 than in 2010. The effect of the different 7-d treatments on the progress of seed filling was nil or negligible at 29/20°C (Fig. 2A), whereas seed filling ended earlier at 34/20°C (Fig. 2C, D), or later at 15/10°C (Fig. 2B). In 2010, T_1_ and T_2_ showed the greatest effects at 34/20°C (Table 2) but with T_2_ reducing final seed weight more than T_1_ (Fig. 2C). In 2011, the 7–65 DAA treatments provided the greatest difference from ambient at 15/10°C (longer filling and greater seed weight) and at 34/20°C (shorter filling and lower seed weight), with T_1_ showing the greatest effect amongst the 7-d treatments at both the extreme temperatures (Fig. 2B, D, Table 2).

**Table 1.** Quantification of seed filling (parameter values of logistic regressions*), harvest maturity (HM) and longevity at HM in spring wheat ‘Tybalt’ in response to temperature

Year	Day/night Temperature (°C)	Treatment	*B* (s.e.)	*M* (s.e.)	*C* (s.e.)	Maximum grain filling rate (mg d^–1^)	Seed filling duration (d)	Final seed dry weight (mg)	Harvest maturity (DAA)	Longevity at HM, *p_50_* (d) (s.e.)
2010	Ambient	Control	0·17 (0·01)	20·27 (0·33)	38·59 (0·38)	1·6	38	39	47	44 (2·5)
	29/20 (°C)	T1	0·23 (0·01)	18·63 (0·30)	36·53 (0·32)	1·8	32	37	47	53 (2·7)
		T2	0·23 (0·02)	18·16 (0·49)	35·43 (0·56)	1·7	31	35	47	27 (0·3)
		T3	0·19 (0·01)	19·62 (0·36)	38·12 (0·42)	1·8	35	38	47	31 (0·2)
		T4	0·18 (0·01)	19·53 (0·52)	36·81 (0·48)	1·7	36	37	47	52 (1·1)
		T5	0·17 (0·01)	20·06 (0·29)	38·11 (0·33)	1·6	37	38	47	52 (1·7)
		T6	0·17 (0·01)	20·05 (0·25)	38·09 (0·29)	1·6	37	38	47	41 (1·1)
	34/20 (°C)	T1	0·29 (0·04)	16·02 (0·53)	30·66 (0·55)	2·2	26	31	47	35 (0·3)
		T2	0·36 (0·04)	14·34 (0·37)	23·44 (0·31)	2·1	22	23	47	48 (0·5)
		T3	0·20 (0·01)	18·96 (0·18)	34·89 (0·20)	1·7	34	35	40	45 (1·3)
		T4	0·19 (0·01)	19·29 (0·22)	36·08 (0·25)	1·7	35	36	47	37 (0·7)
		T5	0·18 (0·01)	19·98 (0·33)	37·99 (0·38)	1·7	37	38	47	52 (1·1)
		T6	0·17 (0·02)	19·95 (0·57)	37·83 (0·65)	1·6	37	38	47	60 (2·5)
2011	Ambient	Control	0·18 (0·02)	23·56 (0·76)	51·87 (1·22)	2·3	40	52	51	28 (0·3)
	15/10 (°C)	7–65 DAA	0·16 (0·01)	24·41 (0·46)	57·90 (0·86)	2·3	43	61	65	14 (0·7)
		T1	0·16 (0·01)	22·32 (0·26)	52·09 (0·43)	2·1	42	58	51	37 (0·4)
		T2	0·15 (0·01)	21·92 (0·51)	52·18 (0·74)	2·1	40	52	51	31 (0·3)
		T3	0·16 (0·01)	23·06 (0·59)	52·80 (0·91)	2·1	41	53	51	35 (0·3)
		T4	0·18 (0·01)	23·53 (0·57)	52·70 (0·92)	2·5	39	53	51	26 (0·3)
		T5	0·17 (0·01)	24·01 (0·49)	53·68 (0·80)	2·3	41	54	51	28 (0·4)
		T6	0·17 (0·01)	23·90 (0·32)	53·21 (0·51)	2·3	41	53	51	24 (0·3)
	34/20 (°C)	7–65 DAA	0·43 (0·09)	13·22 (0·81)	25·85 (0·71)	2·6	21	26	30	37 (0·3)
		T1	0·23 (0·01)	16·27 (0·38)	33·47 (0·39)	2·4	29	33	44	25 (0·4)
		T2	0·23 (0·01)	18·59 (0·33)	40·16 (0·40)	2·4	31	40	51	32 (0·3)
		T3	0·25 (0·01)	18·98 (0·21)	38·99 (0·26)	2·4	31	39	44	36 (0·3)
		T4	0·19 (0·01)	22·65 (0·57)	49·02 (0·86)	2·3	38	49	51	35 (0·3)
		T5	0·18 (0·02)	23·09 (0·72)	50·52 (1·12)	2·3	39	51	51	26 (0·3)
		T6	0·17 (0·01)	23·86 (0·43)	53·16 (0·70)	2·3	41	53	51	40 (0·4)

* *R* ^2^ exceeded 0·988 in all but one regression (0·941, 7–65 DAA, 34/20°C).

**Table 2.** Variation in temporal sensitivity, and in peak temporal sensitivity (in parentheses), and the direction of the effect, amongst wheat seed desiccation, filling or quality parameters to temporary (7 d) exposure to temperatures lower or higher than ambient

Parameter	Effect detected [most-sensitive] period (DAA) (+/–; direction of effect cf. control)			
	2010		2011	
	29/20°C	34/20°C	15/10°C	34/20°C
Seed desiccation period	7–42 [14–21] (–)	7–42 [14–21] (–)	nil*	7–49 [21–28] (–)
Seed filling duration	7–21 [7–21] (–)	7–21 [14–21] (–)	7–21 [7–14] (+)	7–28 [7–14] (–)
Final seed dry weight	14–21 [14–21] (–)	7–21 [14–21] (–)	7–21 [7–14] (+)	7–28 [7–14] (–)
Onset of germinability	7–35 [14–21] (+)	7–35 [7–21] (+)	nil*	7–35 [7–21] (+)
Onset of desiccation tolerance	7–35 [7–21] (+)	7–35 [7–21] (+)	nil*	7–28 [7–14] (+)
Seed longevity at HM	[7–14]^†^ (+) [14–28]^†^(–) [28–42]^†^ (+)	[7–14]^†^ (–) [35–49]^†^ (+)	[7–14]^†^ (+) [42–49]^†^ (–)	[7–14]^†^ (–) [42–49]^†^ (+)
Protein concentration	[7–21]^†^ (+)	[14–21]^†^ (+)	nil*	7–28 [7–14] (+)
Sulphur concentration	nil*	[7–21]^†^ (+)	nil*	7–28 [7–21] (+)
SDS	[42–49]^†^ (+)	7–28 [7–21] (–)	[7–49]^†^ (+)	[7–21]^†^ (–)
HFN	[7–14]^†^ (–)	7–35 [14–28] (+)	[7–49]^‡^ (+)	[7–49]^‡^ (+)

* Nil or limited temporal sensitivity.

† Lesser effect not detected over wider period.

‡ Note that 7–56 DAA at 15/10°C is also greater than control.

HM, harvest maturity; SDS, sodium dodecyl sulphate; HFN, Hagberg Falling number.

**Fig. 3.**
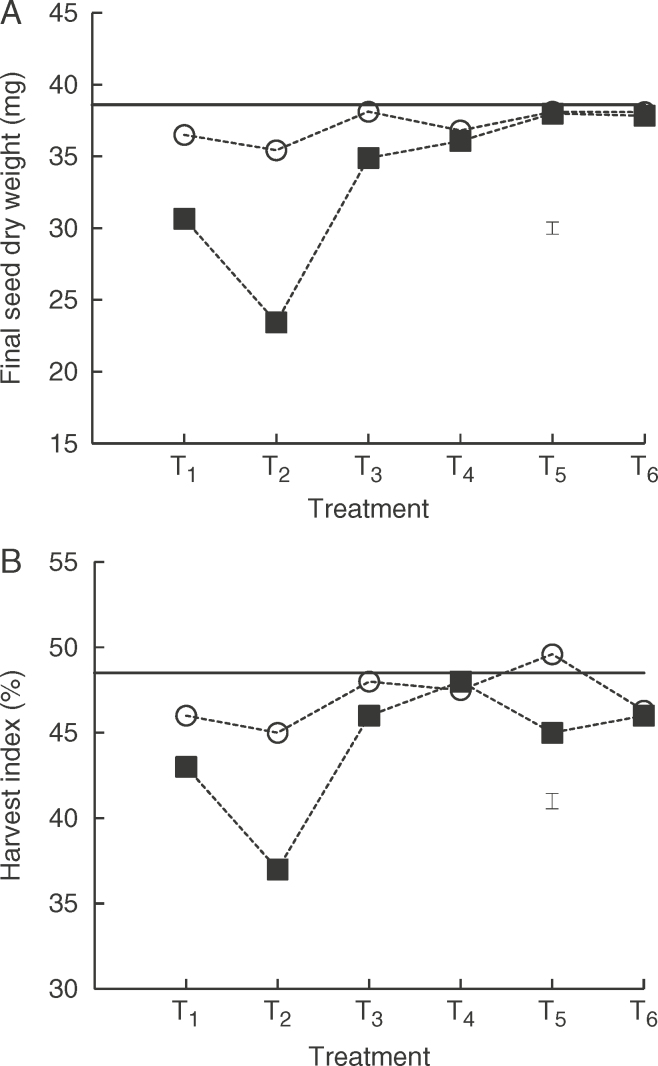
Mean seed dry weight (mg) at maturity (A), from logistic models (Fig. 2; Table 1), and harvest index (%) from independent plants at 61 DAA (B) of spring wheat ‘Tybalt’ control maintained at close to ambient temperature throughout (horizontal line) or exposed for different, successive 7-d periods from 7 DAA onwards (T_1_–T_6_) to day/night temperatures of 29/20°C (○) or 34/20°C (▪) in 2010; vertical lines are standard error of difference.

The additional, independent (cf. Fig. 2) samples of plants harvested at 61 DAA in 2010 showed similar effects of the 7-d treatments at 29/20 and 34/20°C on harvest index as found for seed dry weight (Fig. 3): a small effect of treatment at 29/20°C reducing both in T_1_ and in T_2_ (only), and bigger reductions at 34/20°C with greater effect in T_2_ than T_1_ and with little effect of later treatments (Fig. 3B).

### Ability to germinate

**Fig. 4.**
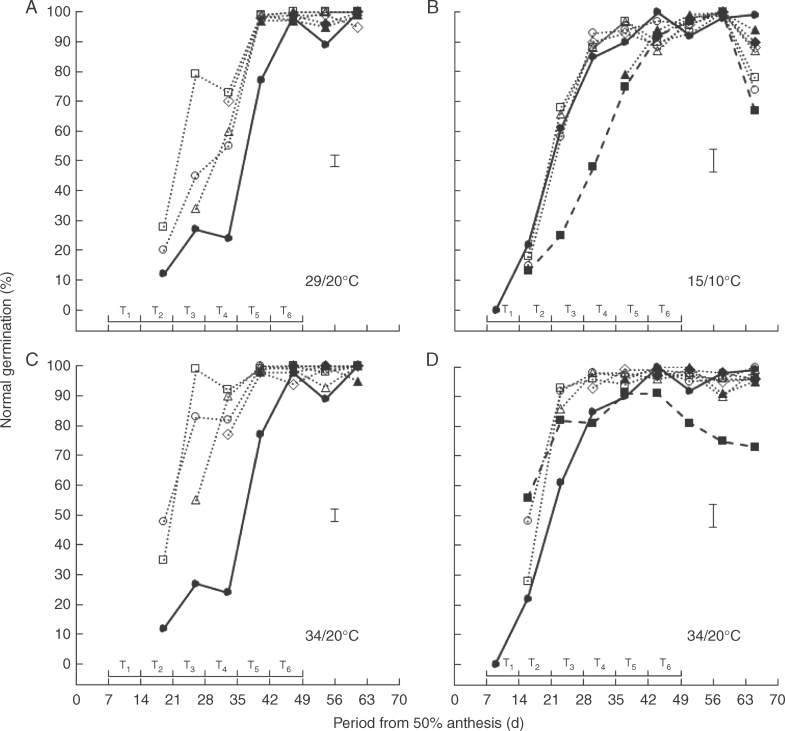
Changes in ability to germinate normally of fresh seed of spring wheat ‘Tybalt’ control plants maintained at close to ambient temperature throughout (**•**) or exposed for different, successive 7-d periods from 7 DAA onwards to day/night temperatures of (A) 29/20°C and (C) 34/20°C (2010) or (B) 15/10°C and (D) 34/20°C (2011); T_1_ (○), T_2_ (□), T_3_ (△), T_4_ (◊), T_5_ (▲), T_6_ (◆). In 2011, treatments were also provided at 15/10°C or 34/20°C from 7 to 65 DAA (◼). The horizontal bars represent the different 7-d periods (T_1_–T_6_) when temperatures higher or lower than ambient were applied; vertical bars are least significant difference (*P* = 0·05).

Temperature, time of treatment, time of harvest, their second-order interaction and almost all first-order interactions amongst them affected percentage germination (*P* < 0·05) for each of fresh and dried seeds in 2010 and in 2011. Ability to germinate *ex planta* developed throughout the 44–47-d period after anthesis in the control, such that almost all treatments provided 100 %, or close to, ability to germinate normally during late seed development and maturation when tested without desiccation *ex planta* (Fig. 4). The exception was the 7–65 DAA treatment at 34/20°C in 2011 where a decline was detected amongst the last four harvests (Fig. 4D).

At temperatures above ambient, the treatments resulted in more rapid development in ability to germinate, whereas this development was delayed at 15/10°C in the 7–65 DAA and T_5_ treatments. Amongst the 7-d treatments at 29/20°C, T_2_ improved in ability to germinate sooner than T_1_ and T_3_ (Fig. 4A) whilst at 34/20°C in 2010 T_1_ and T_2_ were similar with T_3_ slightly delayed (Fig. 4C). Similarly at 34/20°C in 2011, T_1_ and T_2_ were earlier than the ambient control and similar to each other and to the 7–65 DAA treatment (Fig. 4D). In the final (compared with the penultimate) harvest, all treatments at 15/10°C showed a decline in ability to germinate with the 7–65 DAA, T_1_ and T_2_ treatments showing greater reductions than T_3_–T_6_ (Fig. 4B). The 7–65 DAA treatment at 34/20°C in 2011 was the only other example of a decline in ability to germinate being detected and this was progressive throughout the 21-d period ending at the final harvest (Fig. 4D).

**Fig. 5.**
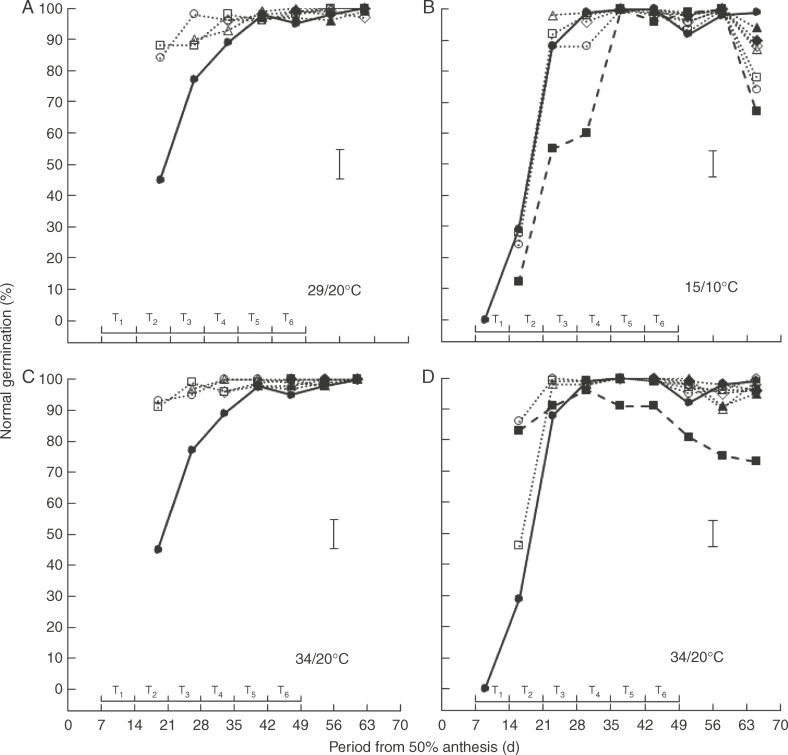
Changes in ability to germinate normally of dried seed of spring wheat ‘Tybalt’ control plants maintained at close to ambient temperature throughout (**•**) or exposed for different, successive 7-d periods from 7 DAA onwards to day/night temperatures of (A) 29/20°C and (C) 34/20°C (2010) or (B) 15/10°C and (D) 34/20°C (2011); T_1_ (○), T_2_ (□), T_3_ (△), T_4_ (◊), T_5_ (▲), T_6_ (◆). In 2011, treatments were also provided at 15/10°C or 34/20°C from 7 to 65 DAA (◼). The horizontal bars represent the different 7-d periods (T_1_–T_6_) when temperatures higher or lower than ambient were applied; vertical bars represent least significant difference (*P* = 0·05).

Comparison of ability to germinate with (Fig. 5) or without (Fig. 4) desiccation *ex planta* showed similar patterns, but with promotion of germination by drying early in development and smaller differences between the 7-d treatments and the ambient control for dried seeds. Nevertheless, advancement of ability to germinate by treatment at 29/20°C (Fig. 5A) and 34/20°C (Fig. 5C, D), delay at 15/10°C (Fig. 5B), and decline in ability to germinate between the penultimate and final harvests for all treatments at 15/10°C (Fig. 5B) and progressively after 30 DAA in the 7–65 DAA treatment at 34/20°C (Fig. 5D) were again detected. The decline in ability to germinate for T_1_–T_6_ at 15/10°C from 58 to 65 DAA, i.e. when all plants had been returned to ambient, for both fresh and dried seeds (Figs 4B, 5B), coincided with a 7-d decline in ambient mean temperature of over 8°C to only 12·5°C at 65 DAA; this is a similar mean temperature to the 7–65 DAA 15/10°C treatment at this time, which also declined in ability to germinate from 58 to 65 DAA.

### Seed longevity

**Fig. 6.**
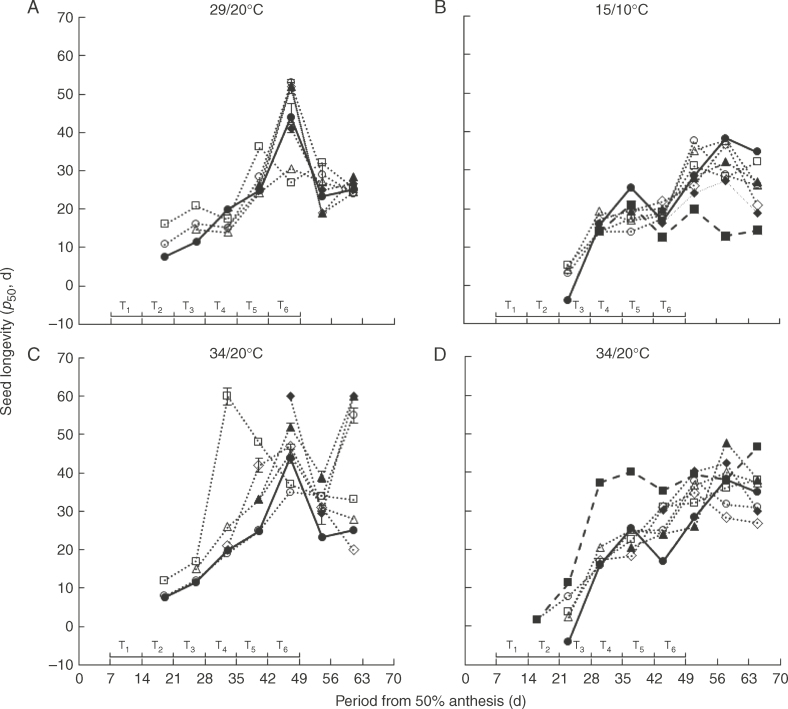
Change in seed longevity (*p*_50_, d) during seed development and maturation of spring wheat ‘Tybalt’ control plants maintained at close to ambient temperature throughout (**•**) or exposed for different, successive 7-d periods from 7 DAA onwards to day/night temperatures of (A) 29/20°C and (C) 34/20°C (2010) or (B) 15/10°C and (D) 34/20°C (2011); T_1_ (○), T_2_ (□), T_3_ (△), T_4_ (◊), T_5_ (▲), T_6_ (◆). In 2011, treatments were also provided at 15/10°C or 34/20°C from 7 to 65 DAA (◼). The horizontal bars represent the different 7-d periods (T_1_–T_6_) when temperatures higher or lower than ambient were applied. Vertical bars show standard errors of the estimates of *p*_50_ where larger than symbols.

Seed survival curves conformed to negative cumulative normal distributions and were quantified by eqn (4). The fitted curves differed in slope (1/*σ*; *P* < 0·001) and intercept (*K_i_*; *P* <0·001), the latter varying in value much more than the former, amongst treatment combinations within each year. Subsequent seed longevity, the period for viability to decline to 50 % provided from the fitted seed survival curves, increased during development and maturation in the ambient controls reaching maximum values 47 DAA (2010) or 58 DAA (2011) and declining thereafter (Fig. 6).

The majority of 7-d treatments at 29/20°C had little effect, except in T_1_ and T_2_ where longevity developed earlier and also maximum longevity was reduced in T_2_ and T_3_ where it failed to achieve the, brief, peak values detected at 47 DAA in the remaining treatments (Fig. 6A). The 7-d treatments at 15/10°C had little consistent effect: in the final two harvests the control provided greater longevity, whereas the 7–65 DAA treatment reached maximum longevity much earlier at 37 DAA with no change over the subsequent 28 d *in planta* and so the poorest longevity (compared with ambient and T_1_–T_6_) from 44 DAA onwards (Fig. 6B).

The 7–65 DAA and some 7-d treatments at 34/20°C resulted in slightly earlier development of longevity (Fig. 6C, D). The pattern was rather more erratic in 2010 (Fig. 6C) than in 2011 (Fig. 6D). At the final harvest in 2010, 14 d after maximum longevity in the control and also 14 d after harvest maturity (Fig. 2), and so representing delayed harvest, differences amongst treatments at 34/20°C were considerable: T_2_–T_4_ provided estimates of longevity similar to the control whereas T_1_, T_5_ and T_6_ were much greater (Fig. 6C). At harvest maturity in 2010, T_1_ and T_4_ provided shorter, T_2_ and T_3_ similar, and T_5_ and T_6_ greater longevity than the control (Table 1). Differences amongst treatments in the final harvest in 2011 were smaller than in 2010, with longevity for 7–65 DAA greater than the control, T_2_, T_3_ and T_5_ similar to the control, and T_1_, T_4_ and T_6_ less than the control (Fig. 6D). At harvest maturity in 2011, 7–65 DAA and T_6_ at 34/20°C provided the greatest longevity (Table 1).

There was a consistent contrast between the effects of the 7-d treatments at different periods at 15/10 and 34/20°C on subsequent seed longevity at harvest maturity. In early seed development, longevity was affected most by treatment at 7–14 DAA and was increased by 15/10°C but reduced by 34/20°C (Table 2). During maturation drying, longevity was affected most by treatment at 42–49 DAA in 2011 but this time it was reduced by 15/10°C and increased by 34/20°C (Table 2). In 2010, the early negative effect of 34/20°C was also greatest 7–14 DAA, but the later greatest positive effect was detected over the wider period 35–49 DAA.

### Bread-making quality

**Fig. 7.**
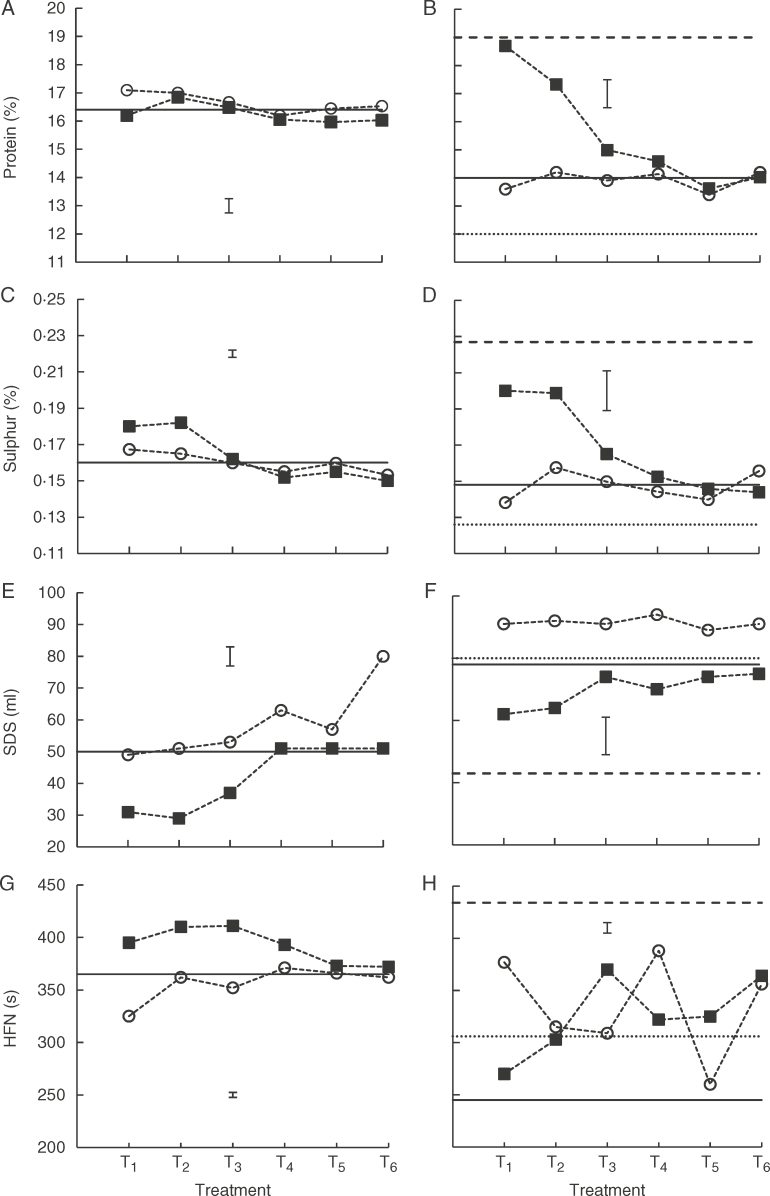
Crude protein concentration (%), sulphur concentration (%), SDS-sedimentation volume (SDS, mL) and Hagberg Falling number (HFN, s) of spring wheat ‘Tybalt’ harvested 61 (2010) or 65 DAA (2011) following exposure for different 7-d periods (T_1_, 7–14 DAA to T_6_, 42–49 DAA) to lower (○) or higher temperature (◼): A, C, E, G, 2010, 29/20°C (○) or 34/20°C (◼); B, D, F, H, 2011, 15/10°C (○) or 34/20°C (◼). Horizontal solid lines show values for the ambient control (—) in each year. Broken lines show values for treatment from 7 to 65 DAA at 15/10°C (…) or 34/20°C (- - -) in 2011. Vertical bars represent least significant difference (*P* = 0·05).

Every aspect of bread-making quality investigated (protein or S concentration, SDS, HFN) was affected by temperature, time of treatment and their interaction (*P* < 0·01) within each year. As would be expected from the greater mean seed weight in the cooler 2011 (Fig. 2), protein and S concentrations were each lower in 2011 than in 2010 for ambient controls (Fig. 7A–D), with SDS greater (Fig. 7E, F) and HFN lower in 2011 (Fig. 7G, H). The 7–65 DAA treatments in 2011 similarly provided lower protein (Fig. 7B) and S concentration (Fig. 7D), greater SDS (Fig. 7F), and lower HFN (Fig. 7H) at 15/10°C (and closer to ambient control) than at 34/20°C.

It was difficult to discern a consistent effect of the 7-d treatments at 34/20 and 29/20°C in 2010 on protein concentration, but both were greater for 14–21 d (T_2_) than ambient whereas later treatment (T_3_–T_6_) provided lower estimates than T_2_ and similar to or lower than the control (Fig. 7A). These trends were more evident for S concentration in 2010, and more so at 34/20°C than at 29/20°C (Fig. 7C). In 2011, these same patterns were evident for both protein and S concentrations at 34/20°C (Fig. 7B, D) with bigger effects than in 2010; no effect of 7-d treatments at 15/10°C was detected for either protein or S concentration.

SDS-sedimentation volumes were largely unaffected by 7-d treatments at 29/20°C in 2010 except for the last (T_6_) (Fig. 7E). There were also no differences in SDS amongst 7-d treatments at 15/10°C in 2011, but all estimates were greater than the ambient control and also than 7–65 DAA at 15/10°C (Fig. 7F). The 7-d treatments at 34/20°C showed the same trend for SDS in both years (Fig. 7E, F): early treatments (T_1_–T_3_ in 2010; T_1_, T_2_ in 2011) lower than the control, with later treatments identical to or approaching the control. In 2011, all 7-d treatments at 34/20°C provided estimates of SDS intermediate between those for 7–65 DAA at this temperature and the ambient control.

Comparison of the 7–65 DAA treatments at 15/10 and 34/20°C in 2011 (Fig. 7H) showed substantial improvement in HFN in the warmer regime. Estimates of HFN from the 7-d treatments in 2010 provided a largely consistent pattern with T_1_–T_3_ at 34/20°C greater than the ambient control but declining thereafter (T_4_) to match control estimates (T_5_, T_6_) (Fig. 7G). The 7-d treatments at 29/20°C provided estimates of HFN similar to the control, except for the lower value for T_1_. In contrast, considerable erratic variation was detected amongst estimates of HFN for T_1_–T_6_ at both 15/10 and 34/20°C in 2011 (Fig. 7H): all those estimates were intermediate between those for ambient and 7–65 DAA at 34/20°C. 

### Variation in temporal sensitivity

Temporal differences in sensitivity to extreme temperature were detected and these periods of greatest sensitivity varied amongst the contrasting criteria investigated (Table 2). Higher temperatures throughout the majority of the period (7 to 42 or to 49 DAA) from anthesis to harvest maturity reduced seed desiccation period with the effect greatest at 14–28 DAA. Seed filling ended well before harvest maturity (Table 1), and so seed filling duration and final seed dry weight were reduced or increased by warming or cooling, respectively, over a more restricted period (7–21 or 7–28 DAA) with greatest effect early in seed filling at 7–14 or 7–21 DAA (Table 2).

Development of ability to germinate and to tolerate desiccation provided broadly similar temporal patterns of earlier increase by higher temperature at 7–28 or 7–35 DAA with greatest sensitivity early in this period at 7–21 or 14–21 DAA, with no effect of lower than ambient temperature detected (Table 2). Discounting results at the closer to ambient 29/20°C, subsequent seed longevity of samples collected at harvest maturity was reduced by higher and improved by lower temperature early in development at 7–14 DAA (Table 2). At each of 15/10°C and at 34/20°C, however, the opposite responses were detected for longevity following exposure late in maturation drying: improved longevity with 7-d exposure to the higher and reduced longevity in the lower temperature at 35–49 or 42–49 DAA (Table 2).

Amongst the bread-making quality criteria, HFN was more affected by extreme temperature for longer and later than protein and S concentrations (with SDS in between the two groups).

## DISCUSSION

Comparison amongst 7–65 DAA treatments at 15/10 and 34/20°C and the ambient control generally showed the expected effects of long exposure to higher temperature throughout wheat seed development and maturation in: reducing seed-filling period (Wiegand and Cueller, 1981) and final seed dry weight (Campbell *et al.*, 1981; Wardlaw *et al.*, 1989; Naylor, 1993); more rapid desiccation (Gooding *et al.*, 2003) and so more rapid development of ability to germinate (Wellington, 1956); more rapid development of ability to survive subsequent air-dry storage (Sanhewe *et al.*, 1996); greater longevity at harvest maturity (Sanhewe *et al.*, 1996); and greater protein and S concentrations (Gooding *et al.*, 2003). The exception was the reduced SDS volume at 34/20°C, which was contrary to the reported increase at higher temperature (Gooding *et al.*, 2003). Hence, opposing effects were detected amongst these yield and quality variables with higher temperature from 7 to 65 DAA. These effects were also detected in some but not all of the 7-d treatments at temperature extremes: that is, temporal sensitivity to extreme temperature post-anthesis was detected.

Mass maturity occurred 22 d earlier in the warmer than cooler 7–65 DAA treatment (Table 1). This effect of increase in mean temperature (11·5°C from anthesis to mass maturity) was 1·9 d °C^−1^, slightly less than the 3·1 d °C^−1^ suggested by Wiegand and Cueller (1981). The overall effect of higher temperatures in reducing durations to mass maturity in turn reduced final seed dry weight (Table 1). The early 7-d treatments at 34/20°C also advanced mass maturity and consequently reduced final seed dry weight (Table 1) with positive linear relations between seed-filling period and dry weight in each year (Supplementary Data Fig. S1), and reduced harvest index (HI) also (Fig. 3). An increase of 0·9–1·2 % HI d^−1^ has been reported during the seed-filling phase under normal fertility conditions (Moot *et al.*, 1996; Wheeler *et al.*, 1996). The control in 2010 provided an estimate of 1·2 % HI d^−1^ and so at the top end of that range. Treatment at 29/20°C and much more so at 34/20°C at 7–14 and 14–21 DAA reduced HI (Fig. 3). The reduction from the ambient control, i.e. the effect of treatment, was 0·5–0·7 % HI d^−1^ and so only around half of the overall estimate in the control. Hence these 7-d treatments were damaging to final seed dry weight, but the effect was less than what would be expected from a constant rate of change in HI. Examination of the 7–14 and 14–21 DAA treatments at 34/20°C (Fig. 2) suggests that seed filling rate increased slightly around this period in both years as a result of the higher temperature, confirmed by the fitted relations in 2010 but not 2011 (Table 1). This compensated in part for reduced durations. This may have resulted from remobilization of dry matter from roots to ears under temperature stress (Ferris *et al.*, 1998). Hence although 7 d at these two warmer regimes reduced seed dry weight, the effect was not as injurious as might be expected.

Drying immature seeds *ex planta* promoted ability to germinate (Figs 4 and 5). Full, or almost full, ability to germinate was first achieved in the dried seed controls at or before mass maturity (38 and 40 DAA in 2010 and 2011, respectively; Table 1). High ability to germinate was subsequently maintained for a considerable period during development and maturation (e.g. 30–65 DAA in 2011, Fig. 5). Seed longevity was a more sensitive indicator of seed quality amongst high-viability samples, as expected (Ellis and Roberts, 1981; Yadav and Ellis, 2016), and continued to improve in the ambient control until 47 DAA in 2010 or 58 DAA in 2011, before then declining (Fig. 6). Maximum longevity was not achieved until 9 d (2010) or 11 d after mass maturity when seeds had dried *in planta* to (47 DAA, 2010; Fig. 1, Table 1), or almost to, harvest maturity (51 DAA, 2011; Fig. 1, Table 1). The benefit of premature desiccation on early ability to germinate, the discrimination in seed quality by longevity amongst high-viability samples, the continued improvement in subsequent seed longevity beyond mass maturity and the achievement of maximum longevity as seeds approach harvest maturity, with possible decline thereafter, all confirm previous research in wheat (Ellis and Pieta-Filho, 1992; Sanhewe *et al.*, 1996; Ellis and Yadav, 2016; Yadav and Ellis, 2016). The decline in longevity in the control from 58 to 65 DAA in 2011 (Fig. 6), after harvest maturity, was matched by a decline in ability to germinate in all the treatments at 15/10°C but not in the ambient control (Figs 4 and 5). This conforms with the poorer longevity in all treatments at 15/10°C, in which the 7–65 DAA treatment failed to improve in longevity beyond 38 DAA, whereas that for the ambient control did improve thereafter (Fig. 6). This plateauing of longevity from 37 to 65 DAA at 15/10°C, i.e. from early in maturation drying until harvest maturity and beyond, matches the circumstance suggested by Yadav and Ellis (2016) where seed deterioration and improvement occur simultaneously with little apparent net change in subsequent longevity with prolonged period *in planta* in less than ideal environments.

This plateauing at 15/10°C, and the comparison with longevity in the 7–65 DAA treatment at 34/20°C, confirms the conclusion of Sanhewe *et al.* (1996) that cool conditions throughout seed development and maturation provide poor, and warm conditions provide much better longevity. Moreover, the current results would extend this conclusion from investigations at mean temperatures from 14·3 to 18·4°C (Sanhewe *et al.*, 1996) to the wider range of 13·3–29·3°C (at 7–65 DAA; or 14·2–25·7°C from anthesis to harvest maturity). However, the results from different 7-d periods at extreme temperatures indicate that the positive relationship between seed longevity improvement and temperature may not apply throughout the entire period (Table 2).

The seed viability equation (Ellis and Roberts, 1980) suggests that all seed lots of a given crop have the same value of *σ* when stored in an identical, constant hermetic environment. Estimates of 1/*σ* (and so *σ*) varied amongst treatments here, however. In the context of exponential relations between seed longevity and storage environment the variation was small. It is also difficult to provide identical seed moisture contents for different seed lots given the accuracy of the procedure (ISTA, 2013). The mean estimates of *σ* were similar amongst years: 4·03 d in 2010 and 4·05 d in 2011. The seed viability constants for wheat (Ellis and Hong, 2007) provide a greater estimate of 6·07 d for *σ* at 40°C with 15·0 % moisture content; 4·03 d would be estimated for a slightly warmer, more moist environment of 42°C with 15·2 % moisture content, for example. This is, we suggest, reasonable agreement in the context of the variation inherent in the analyses which provided the constants for wheat (Ellis and Hong, 2007). Indeed, those constants provided suitable sampling intervals in the current study.

Temperature affects bread-making quality in wheat (Ciaffi *et al.*, 1996; Uhlen *et al.*, 1998; Altenbach *et al.*, 2003; Balla and Veisz, 2007; Labuschagne *et al.*, 2009; Koga *et al.*, 2015). With the exception of SDS volume, the remaining parameters of bread-making quality investigated were all improved by treatment from 7 to 65 DAA in the warmest regime of 34/20°C (Fig. 7). In general, environments that reduce seed weight increase both protein and S concentrations (e.g. Gooding *et al.*, 2003). That was the case here with negative associations between final mean seed dry weight and protein concentration (*r*=−0·70; *P* < 0·01, 2010; *r*=−0·94, *P* < 0·01, 2011) and positive associations between protein and S concentrations (*r* = 0·58, *P* < 0·05, 2010; *r* = 0·97, *P* < 0·001, 2011). The positive effect of long exposure to higher temperature on HFN (compare 2010 and 2011 ambient; and 7–65 DAA at 15/10°C or 34/20°C, Fig. 7G, H) is consistent with earlier studies (Kettlewell, 1997; Kettlewell and Cashman, 1997; Gooding *et al.*, 2003). The suggestion that larger seeds show reduced HFN because they have impaired control over pre-maturity alpha-amylase and hence lower HFN (Evers *et al.*, 1995) was supported by an association in 2010 (*r*=−0·61, *P* < 0·05), but not in 2011 (*r*=−0·32, *P* > 0·05), nor over all samples (*r*=−0·47; *P* > 0·05). This agrees with the report of no mechanistic link between alpha-amylase content and seed size (Farrell and Kettlewell, 2008).

The decline in ability to germinate from 58 to 65 DAA for T_1_–T_6_ and from 7 to 65 DAA at 15/10°C (Figs 4B and 5B), but not in warmer treatments (Figs 4 and 5), coincided with an ambient cold period. The previous cooler exposure may perhaps have left these treatments’ seeds less resilient to the later cool environment. This is speculation, but would be compatible with the positive relationship between temperature and subsequent seed longevity in late development and maturation (Table 2). It would also suggest that cool conditions 7–21 DAA (i.e. T1, T2) provide the poorest resilience to subsequent low temperatures after harvest maturity for ability to germinate.

The positive response of subsequent longevity to temperature detected for 7-d treatments late in maturation (Table 2) is the dominant one for exposure throughout seed development and maturation (i.e. the 7–65 DAA treatments here). The failure of longevity to improve further beyond 37 DAA in the 7–65 DAA 15/10°C treatment supports the possibility of a brief negative response of longevity at harvest maturity to temperature in early seed development. The potentially contradictory consequences for wheat seed longevity at harvest maturity from short exposure to extreme temperature early (negative response to temperature) or late (positive response to temperature) in development and maturation (Table 2) may have parallels in rice (*Oryza sativa*). Rice seed longevity is improved by high-temperature drying *ex planta* for seeds harvested during late maturation drying at moisture contents between 35 and 16 % (Whitehouse *et al.*, 2015). This contrasts with the damaging effects of high-temperature environments *in planta* earlier in development (Ellis *et al.*, 1993; Ellis and Hong, 1994; Ellis, 2011).

The contrasting effects on subsequent seed longevity from short exposure to warm conditions early in development compared to late in maturation might be a consequence of different biochemical pathways, differing in sensitivity to temperature, operating at different periods during seed development maturation. For example, in the dicot *Brassica campestris* (*rapa*) improvement in subsequent longevity was associated positively with the accumulation of a low-molecular-weight, heat-stable protein early in development and maturation, whereas in late maturation a positive association between longevity and accumulation of raffinose series oligosaccharides was found (Sinniah *et al.*, 1998). Similarly, Leprince *et al.* (2017) have shown considerable contrasts between the biochemical pathways active early in seed development and maturation and those active in late maturation in the dicots *Arabidopsis* and *Medicago truncatula*, whilst Lehner *et al.* (2006) suggested that improvement in the tolerance of wheat seed to ageing may be related to raffinose accumulation during maturation drying. Shewry *et al.* (2012) reported a radical switch in the transcriptome profile of wheat at 12–21 DAA, when ability to germinate and tolerate desiccation developed here (Figs 4 and 5). After mass maturity, transcripts continued to be expressed in the embryo but not in the endosperm or pericarp. Sucrose, raffinose, betaine, glutamate, tryptophan, trigonelline and choline increased in the extractable metabolome post-seed filling, and Shewry *et al.* (2012) suggested that betaine, choline and trigonelline accumulating during late seed development and maturation may improve osmoprotection.

Bread-making quality tended to be most affected by brief high temperature treatment early in development and largely in the same period when seed filling duration was most affected (Table 2). High temperature increased protein and S concentrations most when applied 7–14 or 14–21 DAA. This was expected, for protein at least, since nitrogen accumulation in seeds is less adversely affected by high temperature than starch because much of the nitrogen uptake by the plant occurs before anthesis (Sofield *et al.*, 1977; Uhlen *et al.*, 1998; Altenbach *et al.*, 2003; Gooding *et al.*, 2003; Dupont *et al.*, 2006). There was a reduction in SDS volume in both years when plants were exposed to 34/20°C at 7–14 DAA (Table 2). Similarly, Randall and Moss (1990) reported that average daily temperatures above 30°C, even for only 3 d, reduced dough strength. The contradictory effects of higher temperature on increasing protein concentration and reducing SDS volume (indicating better and poorer bread-making quality, respectively) detected here are compatible with reported effects of stress during seed-filling (Gooding *et al.*, 2003). Finally, HFN was increased convincingly by successive 7-d treatments at 34/20°C from 7 to 35 DAA in 2010, and so compatible with the reported effects of long-duration treatment (see above), whereas estimates in (cooler) 2011 were erratic and lower (Fig. 7G, H). Temperature affected HFN for longer than protein concentration (Table 2). This agrees with an analysis of UK crop records in which the positive effect of temperature on protein concentration ended sooner than that on HFN (Smith and Gooding, 1999).

Wheat is important to humankind for sustenance. It is also a model species for research. Our conclusion that different aspects of seed (weight, ability to germinate, longevity, composition, food processing quality, etc.) are not only sensitive to short periods of extreme temperature but also differ temporally (i.e. when during seed development and maturation they show greatest sensitivity, and potentially vulnerability) may apply more widely. Short periods of extreme temperature may affect adaptation (vagile ecology) and competitiveness of wild species, and in other crop plants, value for sowing, human nutrition and food processing. Similarly, the conflicting response of longevity at harvest maturity to brief extreme temperature, negative early in development but positive during maturation, should also be investigated in contrasting species.

In conclusion, higher temperatures advanced development and maturation, as expected, with temporal sensitivity to extreme temperature detected. This varied in the direction of the response as well as in the timing of greatest sensitivity amongst contrasting variables. Temporal sensitivity to reduced production from high temperature was greatest in early-seed filling. Several criteria of bread-making quality also tended to be most sensitive then, with protein and S concentrations improved but SDS volumes damaged by brief high temperature. In contrast, subsequent seed longevity was poorest in response to low temperature (and improved by high temperature) later in development and maturation, with some possibility of improvement by low but damage from higher temperature early in development. Hence, traits sensitive to temperature after mass maturity were associated with the embryo and aleurone layer whilst those associated with the endosperm were particularly sensitive to temperature at early- to mid-seed filling. Temporal sensitivity of wheat quality to extreme temperature variation during development amongst different criteria of quality will need to be accommodated in the global challenge of adapting to and mitigating the consequences of climate change for future food security. 

## SUPPLEMENTARY DATA

Supplementary data are available online at www.aob.oxfordjournals.org and consist of Fig. S1. Positive associations between final mean seed dry weight (mg) and duration of seed filling (d) for spring wheat ‘Tybalt’ control plants maintained at close to ambient temperature throughout (△) or withdrawn from this regime and exposed for different periods to different temperatures [A, 2010 (*r* = 0·983; *P* < 0·001), 29/20°C (○), 34/20°C (◼); B, 2011 (*r* = 0·981; *P* < 0·001), 15/10°C (○), 34/20°C (◼)].

## Supplementary Material

Supplementary Figure 1
