# Supplementary material for: Wheat seed weight and quality differ temporally in sensitivity to warm or cool conditions during seed development and maturation
Source: Ann Bot. 2017 Jun 15;120(3):479–93. doi: 10.1093/aob/mcx074 (PMC5591415; doi:10.1093/aob/mcx074)
Supplement: Supplementary Figure 1 [file mcx074_Suppl_Supplementary_Figure_1.docx]

| Seed dry weight (mg) |  |
| --- | --- |
|  |  |
|  | Seed filling duration (d) |

Supplementary Figure 1
